# Hospitalized care for MDR-TB in Port Harcourt, Nigeria: a qualitative study

**DOI:** 10.1186/s12879-016-2114-x

**Published:** 2017-01-10

**Authors:** Kingsley Lezor Bieh, Ralf Weigel, Helen Smith

**Affiliations:** 1 State TB and Leprosy Control Programme, Rivers State Ministry of Health, Port Harcourt, Nigeria; 2 Liverpool School of Tropical Medicine, Liverpool, UK

**Keywords:** Nigeria, MDR-TB, Patients, Providers, Treatment, Qualitative, Psychological, Stigma, Hospitalised care, Experience

## Abstract

**Background:**

In Nigeria multidrug-resistant tuberculosis (MDR-TB) is prevalent in 2.9% of new TB cases and 14% of retreatment cases, and the country is one of 27 with high disease burden globally. Patients are admitted and confined to one of ten MDR-TB treatment facilities throughout the initial 8 months of treatment. The perspectives of MDR-TB patients shared on social media and in academic research and those of providers are limited to experiences of home-based care. In this study we explored the views of hospitalised MDR-TB patients and providers in one treatment facility in Nigeria, and describe how their experiences are linked to accessibility of care and support services, in line with international goals. We aimed to explore the physical, social and psychological needs of hospitalized MDR TB patients, examine providers’ perceptions about the hospital based model and discuss the model’s advantages and disadvantages from the patient and the provider perspective.

**Methods:**

We conducted two gender distinct focus group discussions and 11 in-depth interviews with recently discharged MDR-TB patients from one MDR-TB treatment facility in Nigeria. We triangulated this with the views of four providers who played key roles in the management of MDR-TB patients via key informant interviews. Transcribed data was thematically analysed, using an iterative process to constantly compare and contrast emerging themes across the data set for deeper understanding of the full range of participants’ views.

**Results:**

The study findings demonstrate the psycho-social impacts of prolonged isolation and the coping mechanisms of patients in the facility. The dislocation of patients from their normal social networks and the detachment between providers and patients created the need for interdependence of patients for emotional and physical support. Providers’ fears of infection contributed to stigma and hindered accessibility of care and support services.

**Conclusion:**

The current trend towards discharging patients after culture conversion would reduce the psycho-social impacts of prolonged isolation and potentially reduce the risk of occupational TB from prolonged contact with MDR-TB patients. Building on shared experiences and interdependence of MDR-TB patients in our study, innovative patient-centred support systems would likely help to reduce stigma, promote access to care and support services, and potentially impact on the outcome of treatment.

## Background

A pragmatic public health approach to tuberculosis control has led to a decline in mortality from the disease worldwide with an estimated 37 million deaths prevented between 2000 and 2013 [1]. However, the emergence of multi-drug resistant TB (MDR-TB) has threatened the progress made in TB control globally [2]. MDR- TB is defined as resistance to Rifampicin and Isoniazid, the most effective first line anti-TB drugs [3]. Globally, there were an estimated 480,000 cases and 190,000 deaths from MDR-TB in 2014 [4]. In Nigeria, 2.9% of new cases and 14% of previously treated TB cases were estimated to have had MDR-TB in 2014 [4].

The latest WHO guidelines stipulate a shorter MDR-TB treatment regimen of 9–12 months under specific conditions, as well as the conventional regimen of 20 months, and recommend models of care based principally on ambulatory/home based care rather than models of care based mainly on hospitalization [5]. The intensive phase of conventional MDR-TB treatment regimen involves daily injections of aminoglycosides as well as orally administered potentially toxic and less effective anti-TB drugs for 8 months and oral medication is continued in the last 12 months of treatment [6]. However, national guidelines for the management and control of MDR-TB in Nigeria are based on previous WHO guidelines and recommend admission of patients into specialised centres until sputum samples are ‘culture negative’ [7]. In practice patients are admitted and confined to one of ten specialized treatment centres in Nigeria for the initial 8 months of intensive chemotherapy [8].

The literature describes patient centred models of care, indicating that home or community based models are favoured by patients, family, community members and health workers [9–11]. Indeed, community based care can be more cost effective [2, 12], provide easier access to treatment, and patients are more likely to be able to seek support from their social network [13, 14]. It enhances the psychosocial support available to patients and enables patients to continue earning a living [10]. HIV/MDR TB co-infected patients might benefit most from this approach. They suffer from severe drug side effects and stigma and need substantial social and emotional support, as shown in India [9].

The available literature suggests that hospital based care in Nigeria promotes adherence to treatment during the intensive phase of treatment [8]. Perspectives of MDR-TB patients are shared in social media [15] and rigorous research on patient views of MDR-TB treatment is limited to their experiences of home based care [9–11]. There is a need to better understand views of hospitalized MDR-TB patients and providers and how this is linked to adherence and accessibility of care and support services for MDR-TB [16], in line with international goals [3].

We therefore aimed to (1) explore the physical, social and psychological needs of hospitalized MDR TB patients, (2) examine providers’ perceptions about the hospital based model and (3) discuss the model’s advantages and disadvantages from the patient and the provider perspective.

## Methods

### Study site

The participants for this study were recruited from the University of Port Harcourt MDR-TB treatment centre. The hospital is operated by the University of Port Harcourt Teaching Hospital with support from the Institute of Human Virology of Nigeria (IHVN) and the National TB Control Programme. The IHVN provides training and supplies with funding from Global Fund to fight AIDS, Tuberculosis and Malaria. MDR-TB patients are diagnosed at selected TB clinics across the country, kept on a waiting list and recalled for admission through phone calls or rarely home visits when bed space is available. Due to limited bed space, approximately 30 patients are enrolled in a treatment cycle. Once most of these patients are confirmed culture negative for MDR-TB in a treatment cohort, no new admissions are made due to concerns about reinfection of the cohort that have almost completed treatment. The head physician performs both administrative and clinical functions. Other doctors are on rotation from the teaching hospital and are not accommodated in the facility for on-call duty. Due to acute shortage of staff, only six nurses run shift duty in the hospital. The caterers are non-resident contractors hired by the administrators. Meals are prepared outside the facility and transported to the centre three times daily.

### Design

We used a qualitative approach to understand patients’ and providers’ experiences of hospital-based care for MDR-TB patients, since this uncovers motivations and values which are shaped by everyday circumstances and culture and helps understand how these influence needs and health behaviour.

Discharged patients of different age, sex, marital status and ethnic origin were purposively selected from facility-based hospital records for two gender distinct focus group discussions (FGDs) comprising six men and six women and 11 in-depth interviews (comprising six men and five women). We included patients who were already culture negative for MDR-TB, had spent at least six months on hospital admission, and were discharged within the preceding 12 months. We selected four healthcare providers who played key roles in managing MDR-TB patients in the facility for key informant interviews. We recruited patients attending clinic for regular appointments. Following their specified appointment, the researcher handed patients an information sheet in a sealed envelope. Non-literate participants were taken to a safe and confidential location in a nearby health centre where the content of the information sheet was explained to them using Pidgin English. Participants were allowed to return home with the information sheet and were encouraged to seek clarification from the researchers through phone calls billed to the researchers’ accounts. Participants were further contacted through phone calls to confirm their willingness to participate in the study and to make appointments for data collection.

### Data collection

Separate focus group discussions for men and women were held at a primary healthcare centre on weekends when the centre was closed for regular work. Each session, which lasted between 45 and 60 min, was audio recorded with a digital recording device. Informed consent to participate and to audio record the discussion was sought and obtained from the participants before the start of each focus group discussion. KB facilitated both focus group discussions using a series of core questions and probing questions when needed to gain understanding of the issues while an assistant researcher took notes and managed the recording device. Once the main topics were covered, the facilitator summarized and gave the participants an opportunity to discuss issues they considered important which were not covered initially. At the end of each focus group discussion, participants were offered refreshment and transport tokens before they returned home.

In-depth interviews were conducted with consenting participants from the FGD in the coming days after the first focus group discussion. However, three males and three females who were not involved in the FGD were also selected for the interviews. In-depth interviews allowed further exploration of issues raised in a more relaxed atmosphere, one to one, to allow participants express their own experiences and perspectives more freely. Furthermore, in-depth interviews provided an opportunity for researchers to explore individual experiences rather than social norms that evolved during treatment. Each interview lasted between 20 and 40 min at the health centre after regular working hours or at participants’ homes at their discretion.

Key-informant interviews were held following the completion of the focus group discussions and most of the in-depth interviews. This allowed the researchers to focus on issues raised by patients in the focus group discussions and in-depth interviews, and obtain provider perspectives and insights. Key informants were given the information sheet at least 48 h before the interview, were given the opportunity to ask questions or clarifications before deciding to participate. Informed consent to participate and to audio record the conversation was sought and obtained at the start of each interview. Each interview lasted for about 30 min.

Data collection took place between May and July 2014. Topic guides, initially developed from the study objectives, were field tested before data collection and modified throughout the data collection process to accommodate emerging themes. Data were collected in English and Pidgin English due to the diversity of local dialects in Nigeria. The population of MDR-TB patients enrolled on treatment in a facility is quite limited, and recruitment of recently discharged patients was challenging. Consequently our sample size is small, even for a qualitative study. However, we are confident that after the eleventh interview and second focus group discussion with this homogenous group, no new insights were forthcoming from participants.

### Analysis

Data were transcribed word for word at the end of each session and the assistant researcher compared the contents with the audio recordings to verify the accuracy of the transcription. Data were analysed using the Framework approach for qualitative data analysis [17]. Each transcript was read three times to identify and highlight key words or phrases across the data set. From the list of key words and phrases, we created an initial coding framework in NVIVO software [18], and used this to label features of interest across all transcripts. Subsequently, coded sections were sorted into themes and sub-themes based on a hierarchy and constantly compared and contrasted to incorporate emerging features from the data set. Finally, data extracts from focus group discussions, in-depth interviews and key informant interviews were compared in a chart to understand the different dimensions of participants’ views and experiences for interpretation and reporting.

## Results

Patients and healthcare providers’ perspectives are described separately to highlight the similarities and differences in their accounts and to give meaning to the full range of experiences of hospitalized care for MDR-TB patients. We identified several themes during analysis; however the main findings reflected the psycho-social impact of prolonged isolation and the coping mechanisms of patients in hospitalized care for MDR-TB.

### Patients’ perspectives on hospitalized care

#### Stigmatization exacerbated by treatment and support services

Most patients talked about being treated differently by friends and family members even prior to hospitalization. For example, patients described being forbidden from sharing cutlery with other household members. In other cases, patients described being systematically alienated from groups in the community as group members distanced themselves during activities. However, some patients deliberately withdrew from family activities, based on erroneous beliefs about disease transmission, to protect loved ones and family members. Consequently, non-disclosure of illness status was more important than coping with the shame arising from disclosure. So they preferred to create false stories or refuse to discuss their illness with peers and associates:

*“I isolated myself from my children and my wife. My plate, cup and sleeping area was different. I did not meet my wife that period. Now, I frequently meet my wife” (IDI, 54, Male)*

*“I could not tell them I have this problem oh. I used to tell them I have chest problem. What kind of chest problem? I said chest problem. I could not tell anybody I have such problem. It’s shameful. Seriously, it’s very shameful so I don’t tell them the real thing” (FGD1, 34, Male)*

*“An illness that will make doctors and nurses to run away, if you tell a non-medic, will they stay with you? It’s just my family members. I don’t even say that. Not even to my friend. We work together, we do things together but I cannot tell them. Not even my girlfriend” (FGD1, 29, Male)*

Most patients we talked to reported that it was a rare event for a health worker to enter the wards or have a conversation with them due to fears of infection with MDR-TB. This fragmented interaction hindered service delivery and contributed to deleterious health outcomes in some instances. Some participants recalled that healthcare providers in other facilities, which patients visited for specialised services such as audiometry and chest X-ray avoided contacts with MDR-TB patients and were more resentful than the healthcare providers at the MDR-TB treatment centre.

*“When they said MDR-TB patients are coming, they started running away from us and we felt bad” (FGD1, 29, Male)*

*“Those people condemned us…. They don’t come close to our hostel. They don’t come close to us. We were like untouchable. If we touch something, they can’t touch it. We told them that we lived with people and they didn’t get sick. So why will you be infected in this place” (FGD1, 56, Male)*

*“Even some nurses and medical workers treated us like we are not fit to live again. They keep a distance when they want to communicate with us. If you come closer, they will shout go! go!! go!!! …….. The feeling of stigma is very difficult. I felt like the worst person on earth having MDR-TB” (IDI, 29, Male)*

Most participants found the mandatory use of facemasks in the facility distressing and inconvenient especially at bed times. In some cases, they felt it was derogatory and unfair for patients to use an inferior quality face mask while healthcare providers had a more superior type. There were also reports of healthcare providers castigating patients for not wearing facemasks, which was humiliating for some patients in the facility:

*“It was very difficult the first time but we had to do it. We wore face mask always and the nurses… even though you are far from them, once they see you move the mask down to your nose or under your nose, they will abuse and embarrass you like you are nobody [unimportant]”(IDI, 32, Male)*

*“It is an inferior face mask. It is not a good type. It is the type they are selling in the market that they brought to us. They were using the better type. You see Nigerians! We are supposed to use the good type so that we won’t expose ourselves, but they are using the better type and gave us the fake type. The good type is for doctors and nurses while the inferior type is for the patients. I argued with them seriously. They said, I argue too much because I am educated” (IDI, 54, Male)*

#### Feeling of imprisonment

Living in the hospital was likened to imprisonment by most patients we talked to. According to one patient, he initially feared they were isolated for extermination as a way of preventing them from spreading MDR-TB in the community. Another described feeling depressed to the extent he had had suicidal thoughts. Others expressed feelings of deep anger sometimes targeted at themselves or other patients, but mostly at healthcare providers. These feelings were attributed to the confinement and restriction of movement enforced by healthcare providers in the facility:

*“They seized our movement. They don’t have regard for us. We are just like people in prison. That’s what I meant by imprisonment. They treated us the way they liked, not according to the instruction giving to them” (IDI, 56, Male)*

*“I was not comfortable in that environment. I’m the kind of person that likes working, get something doing but you now confined me to a place, I got bored. Most times, I was angry with myself. I wanted to leave that place. Sometimes, I will just go and sit on my own” (IDI, 29, Female)*

*“It made me to break bottle to fight. It made me to abuse the nurses. It made me to see them like nobody [unimportant]. I got angry with everybody. There were times the doctor would come to calm me down. But I saw everybody as my enemy because that is not a place to stay. These things were upsetting me……. you are just like a prisoner in fact, the prisoners are even better than us. Life was just stagnant” (IDI, 34, Male)*

#### Restricted communication and isolation disrupts patients’ personal relationships

Participants resented the prolonged isolation during hospitalization that denied them the opportunity to socialize with family and friends. Consequently, relationships became estranged in some cases and the resultant psychological effect triggered attempts by patients to leave the facility at the expense of continuous treatment, to repair their fragmented relationships. In some cases, the disruption of interpersonal relationships arose from health care providers’ deliberate attempt at reducing hospital visits. Patients gave examples of how health care provider’s disseminated information that promoted fear of contracting the disease among relatives and peers during counselling:

*“Staying there for 8 months without my children, without my family, all alone, it wasn’t easy because you don’t know what will happen. You just hand over the children to the hands of God and the hands of a stranger. It’s not too good for me as a woman” (IDI, 36, Female)*

*“If I can lose that my lovely Angel because of this treatment, the girl I cherished so much, my next of kin…. they told her, she will be the next to receive treatment if she continued visiting and the relationship scattered [ended]. Where is my happiness? There is no joy in my life again so I can do anything” (IDI, 32, Male)*

*“Even members of my church that brought something for me…. After they told them they will contract this sickness, the people ran away and never came back” (FGD1, 57, Male)*

#### Fellow patients as source of physical and emotional support

Most participants described how they took turns to clean the wards, wash bedding and clothes, and fetch water from neighbouring compounds due to staff shortages, fears of infection among cleaning staff and unstable power supply. Patients explained that they became physically tired from performing these duties. Female patients particularly felt they needed psychological support during hospitalization due to the impact of long term isolation. For most patients, the most important source of physical and emotional support was their fellow patients. Many described how older patients would counsel and console the younger ones as they would their own family members and in turn would receive support for physically demanding tasks. The only support provided by the facility seemed to be frequent health talks highlighting infection control measures delivered by healthcare providers. Patients reported that personalized professional counselling was unavailable and they could not attend or value the health talks because they mistrusted healthcare providers. The mistrust arose from alleged insensitivity of healthcare providers to the privacy needs of patients as confidential information in patients’ hospital records were often disclosed by some healthcare-providers. The reservation stemming from allegations of breach in confidentiality was exemplified by a female participant who said; everyone knew which patient was HIV positive because the nurses talked about it in the open.

*“We were asked to go outside and fetch water. We went outside to fetch water (laughs softly). Those that lacked strength asked us for help. Some of them couldn’t carry water….. Some of them couldn’t do anything for themselves; we were helping them to do some things which we were not supposed to do” (FGD2, 29, Female)*

*“I cried all the time in that hospital because I left school to come for treatment at my young age. This sickness has kept me in this terrible condition, and then Aunty Jane [fellow patient, not real name] will console me saying, “you are still alive, God will heal you… there is hope. Some people are dead because of this illness, some died without getting sick, but you are alive” (IDI, 19, Female)*

*“I continued to think of my family but that lady! [a younger female patient] You see, God brought her for me and she helped me, took care of me and treated me like her mother” (IDI, 58, Female)*

#### Meeting needs through organized group action

Patients described how they organized themselves into a social group with elected leaders during their stay in the facility. These leaders mostly communicated their collective views on a subject to the appropriate authorities. For instance, they presented the group’s recommended food menus to the authorities to resolve their disapproval of the type and quality of food served by food contractors in the facility. In addition to weekly worship meetings, patients met frequently to provide support to each other and to discuss challenges with treatment and support services. Incentives such as stipends were TB control programme initiatives aimed to provide economic support to patients during their stay in the facility. Sometimes, patients organized group protests to draw the attention of the authorities to delays in payment of these stipends as well as to demand for improved services in the facility. According to some patients, these protests were in form of hunger strike or refusal to take medications:

*“As the chairman of the group, I called for a meeting to discuss the situation of the food they gave to us in that hospital” (FGD1, 34, Male)*

*“Sometimes, we had general meetings to encourage ourselves. Even there was a time they were supposed to pay us some money, but they refused to give us. We had to do demonstration and things like that to get something” (FGD2, 29, Female)*

*“Once we rioted over so many things and I was at the front. We said no body will take any food or treatment there, let’s see what will happen. We raised our voice together, and then they called some persons to come and settle the matter” (FGD1, 34, Male).*

### Healthcare provider perspectives on caring for hospitalized patients

#### Stigma linked to fears of infection among non-MDR-TB healthcare providers

Some healthcare providers gave their interpretations of stigma of MDR-TB patients during hospitalization. Although one healthcare provider perceived that the mandatory use of facemasks may have been stigmatizing for patients, others were mostly unaware of the humiliation felt by MDR-TB patients as a result of their infection control practices. However, the general opinion among healthcare providers was that both MDR-TB patients and personnel were frequently stigmatized by other healthcare providers during their regular visits to other facilities for audiometry and chest x-ray. Two healthcare providers explained that fears of infection among other healthcare providers at these facilities resulted in deliberate cancellation of hospital appointments to prevent exposure to MDR-TB from contact with MDR-TB patients.

*“The areas we had challenges were radiography and audiometry because we were depending on others. At a place outside the treatment facility, even the stigma and mind-set of other health care workers to TB is a challenge so they find an excuse not to get it done” (Key informant 2)*

*“If any of them want to ask me something, they gave me facemask, but my workers I tell them not to go close to them. We don’t go close to them. I don’t know if the air I breathe is infected but I pray to God that nothing will happen to me (Key informant 3)*

*“Well, to some extent the use of facemask is stigmatizing, but infection control is a priority” (Key informant 2)*

*“We don’t go into their rooms and patients were not happy about this, they come outside, and you take the vital signs except those that came in very ill. Those ones, you go in to see them but not often” (Key informant 1)*

#### Psychological trauma associated with socio-economic disruption

All the healthcare providers we interviewed reported that some patients were frequently agitated and confrontational in the face of unmet socio-economic needs. In some instances, patients would request to leave the facility for a specified period to attend to a family need. Although these requests were often warranted, they could only empathise with the patients due to the strict hospitalisation policy at the facility. They explained that, once this demand was denied, some patients attempted to temporarily leave without authorisation, occasionally became verbally abusive or refused medications in protest.

*“We had someone who lost his father and felt bad that he wasn’t there to help or attend the funeral. There was a lady whose kids were in school and there was a landmark educational achievement and she couldn’t attend the event. These were some other cases; a newly married couple who just got married but the wife was an in-patient and they were both worried about disclosure to the extended family considering the cultural implication. He had to find a way to explain the absence of his wife. A lady was worried because she felt her absence from home will give room for her husband to engage in extramarital affairs…..so those are the kind of challenges you see” (Key informant 2)*

*“The isolation causes problem and it affects us too because they complain about many things…. They worry about going home when they are in need. Sometimes they reject their drugs, saying they cannot take again” (Key informant 1)*

*“One of them sneaked out of the facility to watch football and the person on duty [healthcare provider] was not happy so they exchanged words [had a quarrel]. So when I came on morning duty, she complained to me about how rude the patient was to her” (Key informant 4)*

*“They demanded a lot asking for money and the rest because many of them are breadwinners in their family, and they asked for money to send to the people they are feeding at home. So that became a problem so.”(Key informant 1)*

#### Perceptions of hospital services

Healthcare providers described how they took extra shifts to compensate for staff shortages in the facility. They were frustrated by the inability of the authorities to pay some allowances for the extra shifts. However they explained that their resentment of the conditions of service did not significantly affect the discharge of their duties. In their opinion, poor service delivery was rather caused by infrequent power supply and lack of critical care equipment such as oxygen cylinders. What they found more disheartening was that they were often held responsible for the problems with service delivery as patients could not distinguish between healthcare providers and hospital management.

*“Sometimes they take it out on the health workers because, they are the people they see. If there is any form of dissatisfaction with service, if there is a power outage, of course they take it out on you and its very painful… you are not providing power, you are making us uncomfortable” (Key informant 2)*

*“If there is oxygen we could use it. They brought an automated oxygen machine, where is the light [electricity] to plug the oxygen concentrator?” (Key informant 1)*

*“Because we are short staffed, we are supposed to run 3 shifts but we are running 2 shifts, so it’s a burden on us. You leave your house 6:30 am and you close by 5 pm. That’s if the person taking over from you comes at 5 pm. By the time you hand over and return home, you are already worn out and they are not paying for the extra time” (Key informant 4)*

## Discussion

To our knowledge, this is the first study that analyses insights of purposively selected patients and key-informants on hospitalized MDR TB care. As such, our findings provide a starting point for further research as they may only scratch the surface of the psycho-social impact of hospitalisation due to potential distortion by the indignity of disclosure and the tendency to present self as auspiciously as possible. The dislocation of patients from normal social networks and the detachment between healthcare providers and patients created the need for interdependence of patients on each other for emotional and physical support. The behaviour of healthcare providers, imposed by the fears of infection, contributed to discrimination of patients in the facility. However, healthcare providers seemed unaware of the stigma felt by patients in the facility as they rather attributed this to the attitude of non-MDR-TB healthcare providers towards patients when visiting other facilities for specialized services. There are potentially many other stories and striking incidents that were never discussed by participants.

This study illuminates how the fears of infection contributed to the stigma of MDR-TB, and in some cases discriminatory behaviour towards patients. At the community and family level, isolation of patients was either self-induced to protect family members or imposed by others. In both instances, stigma was rooted in ignorance of MDR-TB transmission mechanisms as the misperception of possible sexual and faecal-oral transmission of MDR-TB influenced attitude and behaviour. On interaction with healthcare providers, the ability to conceal their diagnosis was lost and patients felt increasingly vulnerable to stigmatization. Healthcare providers purposefully had minimal contact with patients, enforced mandatory use of facemasks in the facility, and patients took on the physical work of cleaning wards and fetching water because cleaning staff feared becoming infected; while most patients referred to these behaviours as ‘stigma’, they are deliberate actions to avoid MDR-TB patients and therefore more accurately described as discrimination. In a qualitative study in Nepal, TB patients also reported being stigmatized by healthcare providers [19]. Furthermore, as HIV is also a stigmatizing disease [9], HIV co-infected MDR-TB patients may have experienced more stigma from fellow patients and healthcare providers due to concerns about breach of confidentiality in the facility. The increased potential for occupational TB among healthcare providers who have prolonged contact with drug-resistant tuberculosis patients has been described [20, 21]. More so, in areas with inadequate infrastructure and poor infection control, healthcare providers’ fears of infection may not be totally unwarranted or misplaced [21]. However the impacts of these fears, often manifest in discrimination, on the emotional and physical wellbeing of patients could undermine MDR-TB control initiatives.

The substantial interdependence among patients to support each other emotionally developed naturally in our case but could be further exploited as an opportunity to improve outcomes. This finding resonates with a recent Medecins Sans Frontieres (MSF) project which identified the role of shared experiences via a social media blog in promoting social support for patients and ensuring adherence to MDR-TB treatment [15]. However, social media blogging might be an insufficient strategy for providing substantial social support especially in low resource settings with mostly non-literate patients and poor internet facilities. Regardless, the prospect for social support should be distinguished from the discriminatory and physically stressful roles assumed by patients in our study, as they strived to mitigate the shortcomings in service delivery, helping each other to cope with strenuous physical activities, such as cleaning toilets, fetching water and washing beddings, at the expense of their physical wellbeing.

Prolonged isolation induced feelings of fear, anger, self-blame, depression and suicide in some of the patients. These feelings have been demonstrated in studies conducted on incarcerated individuals [22, 23]. Indeed, it appears that our patients perceive their situation as imprisonment. However, second line anti-TB drugs may have the potential to cause similar psychological effects [24]. In reality, the contribution of each to the patients’ mental status is difficult to distinguish. The dislocation of patients from normal social networks created the need for interdependence on others within a new social group. This new social identity with fellow patients was formed and reinforced through collective experience of illness enhancing understanding and similarity with new social relationships. Even though nurses are providers of emotional support for patients in hospitalized care [25], mistrust arising from allegations of breaches in confidentiality and the stigma imposed by fears of infection limited their roles in the facility. Within the new social construct, the trust for confidentiality between younger and older females was perceived to be a mother and child relationship. In community-based care, trust for confidentiality may be vested in spouses [9] and religious leaders especially in low resource settings. However, restriction of visits and the remote location of the facility undermined the role of these individuals in providing substantial emotional and psychological support for patients in hospitalized care.

Patients became actors to change their situation. They drew the attention of the authorities to perceived irregularities through organized group protests in form of hunger strike or refusal to take medications. This active and antagonizing role of patients is different from the passive role assumed by hospitalized cancer patients in Kenya [26]. Possibly, MDR-TB patients, on symptomatic recovery did not view themselves as sick but as a community of individuals with similar needs that should be addressed. Communality of behaviour evolves naturally with the new value system of a stigmatized group. Transformation from unexpressive individuals in the larger society to highly vocal individuals occurs within their own social group and commonly, they express themselves in institutional terms [27]. Although cancer patients are hospitalized, they are not isolated and as such receive emotional support from family members. Thus, their perception of services and their reaction to the treatment experience could be different from those of MDR-TB patients in hospitalized care.

With growing evidence that effective treatment expeditiously renders MDR-TB patients non-infectious [28] there is little reason to keep people who are on treatment in hospital for months after they become culture negative. This is especially the case in Nigeria and other countries with limited specialist facilities that operate a waiting list for patients diagnosed with MDR-TB; those waiting to start treatment are highly infectious and can perpetuate community transmission of MDR-TB. In addition to this, the findings presented here show that patients suffer distress, discomfort and other psycho-social impacts when hospitalised for prolonged periods. Recent research from South Africa also suggests that hospitalisation increases treatment costs for MDR-TB [29]. The current practice towards reduction in the duration of hospitalization with a target to discharge patients after culture conversion would reduce the psycho-social impacts of prolonged isolation and potentially reduce the risk of occupational TB from prolonged contact with MDR-TB patients. However, concerns about shortage of healthcare providers to sustain community/home-based care might prolong the proposed transition in Nigeria and similar settings. The identification and implementation of effective infection control interventions that reassure and protect health care providers and are acceptable to patients could dispel the fears of infections, reduce stigma and discrimination and possibly improve patients care. Building on shared experiences of illness and patients’ interdependence in this study, future research could focus on innovative patient-centred support mechanisms that are accessible and incorporate stigma reduction activities and emotional support for patients to improve treatment and support for MDR-TB patients. Further studies should explore the economic impacts of prolonged hospitalization.

## Conclusion

MDR-TB patients who are isolated for treatment in hospitals experience psycho-social trauma due to detachment from their normal social networks. The fears of infection among providers contribute to discrimination of MDR-TB patients and promote stigma, which impact on health services delivery. In the Nigerian context and similar settings which operate a waiting list for admission of MDR-TB patients to treatment facilities, reduction in the period of hospitalization or adopting the more globally preferred home-based model of care for MDR-TB may have benefits for patients and the health system, including reduction in community and nosocomial transmission of MDR-TB, as well as improved social and psychological support for MDR-TB patients. The substantial interdependence of patients on each other for emotional support which evolved as a consequence of the fragmented relationship between providers and patients in our study presents an opportunity for developing innovative patient-centred support systems for MDR-TB management.

## Abbreviations

FGD: Focus group discussion
HIV: Human immunodeficiency virus
IDI: In-depth interview
IHVN: Institute of Human Virology of Nigeria
MDR-TB: Multidrug-resistant tuberculosis
MSc: Master of Science
MSF: Medecins Sans Frontieres
TB: Tuberculosis
UK: United Kingdom
WHO: World Health Organization

## References

[1] World Health Organization. Global Tuberculosis Report 2014. Geneva: World Health Organization; 2014.

[2] Falzon D, Jaramillo E, Wares F, Zignol M, Floyd K, Raviglione MC (2013). Universal access to care for multidrug-resistant tuberculosis: an analysis of surveillance data. Lancet Infect Dis.

[3] World Health Organization (2011). Towards universal access to diagnosis and treatment of multi-drug resistant Tuberculosis:WHO progress report 2011.

[4] World Health Organization (2015). Global tuberculosis report 2015.

[5] World Health Organization (2016). WHO treatment guidelines for drug-resistant tuberculosis 2016 update.

[6] World Health Organization (2011). Guidelines for the programmatic management of drug resistant Tuberculosis 2011 update.

[7] NTBLCP (2011). Guidelines for the clinical management and control of drug resistant Tuberculosis in Nigeria.

[8] Oladimeji O, Isaakidis P, Obasanya OJ, Eltayeb O, Khogali M, Van den Bergh R (2014). Intensive-phase treatment outcomes among hospitalized multidrug-resistant tuberculosis patients: results from a nationwide cohort in Nigeria. PLoS ONE.

[9] Isaakidis P, Rangan S, Pradhan A, Ladomirska J, Reid T, Kielmann K (2013). ‘I cry every day’: experiences of patients co-infected with HIV and multidrug-resistant tuberculosis. Trop Med Int Health.

[10] Horter S, Stringer B, Reynolds L, Shoaib M, Kasozi S, Casas EC (2014). “Home is where the patient is”: a qualitative analysis of a patient-centred model of care for multi-drug resistant tuberculosis. BMC Health Serv Res.

[11] Morris MD, Quezada L, Bhat P, Moser K, Smith J, Perez H (2013). Social, economic, and psychological impacts of MDR-TB treatment in Tijuana, Mexico: a patient’s perspective. Int J Tuberc Lung Dis.

[12] Shin S, Furin J, Bayona J, Mate K, Kim JY, Farmer P (2004). Community-based treatment of multidrug-resistant tuberculosis in Lima, Peru: 7 years of experience. Soc Sci Med.

[13] Brust JC, Shah NS, Scott M, Chaiyachati K, Lygizos M, van der Merwe TL (2012). Integrated, home-based treatment for MDR-TB and HIV in rural South Africa: an alternate model of care. Int J Tuberc Lung Dis.

[14] Nathanson E, Nunn P, Uplekar M, Floyd K, Jaramillo E, Lönnroth K (2010). MDR tuberculosis—critical steps for prevention and control. N Engl J Med.

[15] Horter S, Stringer B, Venis S, du Cros P (2014). “I Can Also Serve as an Inspiration”: A Qualitative Study of the TB&Me Blogging Experience and Its Role in MDR-TB Treatment. PLoS ONE.

[16] Bassili A, Fitzpatrick C, Qadeer E, Fatima R, Floyd K, Jaramillo E (2013). A Systematic Review of the Effectiveness of Hospital-and Ambulatory-Based Management of Multidrug-Resistant Tuberculosis. AmJTrop Med Hyg.

[17] Ritchie J, Lewis J, Nicholls CM, Ormston R. Qualitative research practice: A guide for social science students and researchers. Sage; 2013.

[18] Welsh E (2002). Dealing with data: Using NVivo in the qualitative data analysis process. Forum Qualitative Sozialforschung/Forum: Qualitative Social Research.

[19] Baral SC, Newell JN, Karki DK (2007). Causes of stigma and discrimination associated with tuberculosis in Nepal: A qualitative study. BMC Public Health.

[20] Baussano I, Nunn P, Williams B, Pivetta E, Bugiani M, Scano F (2011). Tuberculosis among health care workers. Emerg Infect Dis.

[21] Von Delft A, Dramowski A, Khosa C, Kotze K, Lederer P, Mosidi T (2015). Why healthcare workers are sick of TB. Int J Infect Dis.

[22] DeVeaux MI (2013). Trauma of the Incarceration Experience, The. Harv CR-CLL Rev.

[23] Haney C (2003). The psychological impact of incarceration: Implications for post-prison adjustment.

[24] Blumberg HM, Burman WJ, Chaisson RE, Daley CL, Etkind SC, Friedman LN (2003). American Thoracic Society/Centers for Disease Control and Prevention/Infectious Diseases Society of America: treatment of tuberculosis. Am J Respir Crit Care Med.

[25] Chalco K, Wu DY, Mestanza L, Munoz M, Llaro K, Guerra D (2006). Nurses as providers of emotional support to patients with MDR-TB. Int Nurs Rev.

[26] Mulemi BA (2008). Patients’ perspectives on hospitalisation: Experiences from a cancer ward in Kenya. Anthropol Med.

[27] Goffman E (1963). Stigma. Notes on the Management of Spoiled Identity.

[28] Dharmadhikari AS, Mphahlele M, Venter K, Stoltz A, Mathebula R, Masotla T (2014). Rapid impact of effective treatment on transmission of multidrug-resistant tuberculosis. Int J Tuberc Lung Dis.

[29] Sinanovic E, Ramma L, Vassall A, Azevedo V, Wilkinson L, Ndjeka N (2015). Impact of reduced hospitalisation on the cost of treatment for drug-resistant tuberculosis in South Africa. Int J Tuberc Lung Dis.

